# Achieving High‐Density and Stress‐Resilient Maize Breeding Via Germplasm Innovation

**DOI:** 10.1002/advs.202600030

**Published:** 2026-06-09

**Authors:** Xinlong Li, Yongqiang Chen, Dong Ding, Xuehai Zhang, Xuxiang Jia, Zhanhui Zhang, Yafei Wang, Weihua Li, Hui Zhang, Jihua Tang

**Affiliations:** ^1^ State Key Laboratory of High‐Efficiency Production of Wheat‐Maize Double Cropping/College of Agronomy/The Shennong Laboratory Henan Agricultural University Zhengzhou China

**Keywords:** four‐step modern breeding pipeline, high‐density planting, individual productivity, maize, stress resilience

## Abstract

Given the global population growth and climate change, feeding the future 10 billion people has become an urgent challenge. As a worldwide crop, maize is pivotal in meeting this demand. Increasing planting density has long been regarded as an effective approach to enhancing maize yield in most production regions. In this perspective, we propose to increase planting density by optimizing plant architecture and balancing population‐level and individual‐level advantage, while also improving individual productivity by optimizing yield components, ideal ear architecture, and enhancing photosynthetic efficiency. Gene pyramiding has been proposed to enhance stress resistance, together with reinforcing lodging resistance, and shifting to earlier diurnal flower opening time to escape heat stress. Additionally, improving nutrient use efficiency can reduce fertilizer dependence, while increased photothermal insensitivity can broaden ecological adaptability. To achieve these objectives, we outline a four‐step modern breeding pipeline integrating variation generation, selection, fixation, and genomic selection for hybrid prediction.

## Graphical abstract

Global population growth and climate change have exacerbated the global food crisis. This perspective presents a conceptual framework focusing on enhancing population advantages. Several novel breeding objectives are proposed to improve density tolerance and stress resistance for yield improvement. Furthermore, we propose the four‐step modern breeding pipeline and discuss how to address trait trade‐offs through a three‐step roadmap.

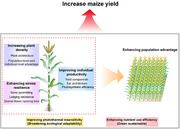

## 1 Introduction

Maize, wheat, and rice collectively supply over 40% of the global caloric intake derived from staple cereals [1]. In parts of Africa and Mesoamerica, maize alone provides over 20% of food calories [2]. Global population growth has been accompanied by a steadily increasing demand for food [3, 4]. Despite this rising demand, widespread hunger remains an unresolved and pressing global challenge. In 2024, an estimated 733 million people worldwide continued to experience hunger, with undernourishment in Africa reaching 20.4% [1], underscoring food security as one of the most serious challenges confronting humanity. From 2003 to 2019, the global cultivated land area expanded by approximately 9%, yet per capita arable land declined by nearly 10% due to population growth [5]. Together, these trends highlight the urgent need for innovative maize breeding objectives and methods to address the global food security challenges.

A central dilemma in maize breeding is the inherent trade‐off between individual productivity and population‐level yield. In practice, historical yield improvements in maize have been closely associated with increases in planting density. Between 1930 and 2010, maize planting density in the United States increased from approximately 30 000 to 80 000 plants per hectare, coinciding with more than a sevenfold increase in grain yield [6, 7]. In contrast, per‐plant yield exhibited only minimal improvement over the same period [8]. Similarly, the China‐wide farmer‐plot study supports continued density improvement, but not in the simple sense that “more plants are always better”: the gains are regional and depend heavily on management, with national density increasing from about 54 300 to 62 200 plants ha^−1^ and current regional means ranging from about 49 574 to 69 921 plants ha^−1^ [9]. However, the recent U.S. Midwest study points in a different direction. It reports that optimum density has largely stabilized after 2010, while yield gains now come more from higher yield per plant [10]. The density‐driven yield strategy exhibits divergent regional applicability: it remains effective for yield improvement in most Chinese maize‐growing regions, yet reaches a practical upper limit in the U.S. Midwest, where future yield gains rely primarily on elevated individual productivity. Collectively, these observations underscore that planting density cannot be increased indefinitely, and further population yield gains at the stabilized optimal density depend on the improvement of individual productivity to enhance population advantages.

Climate change poses a significant threat to global ecosystems and socioeconomic development, with profound consequences for agricultural productivity. Under ongoing global warming, both the frequency and intensity of extreme weather events have increased, with heat stress and flooding causing recurrent reductions in crop yields [11, 12, 13]. Since 1980, climate change has resulted in estimated yield losses of 3.8% in global maize production and 5.5% in wheat production [14]. Mitigating the impacts of climate change on food production has therefore become an urgent challenge, and the development of crop varieties with enhanced resilience has emerged as a key strategy for safeguarding food security.

In this perspective, we outline a conceptual framework focused on enhancing population advantage through increasing planting density, improving individual plant productivity, and enhancing stress resilience to achieve sustained yield improvement in maize. We propose that planting density can be increased through the optimization of plant architecture and a balance between population‐level and individual‐level advantages. At the population level, individual plant productivity under high‐density population conditions can be further improved by optimizing yield components, ideal ear architecture, and enhancing photosynthetic efficiency. We also define a set of stress‐resistance objectives, including the leveraging gene pyramiding, reinforcement of lodging resistance, and the advancement of earlier diurnal flowering to mitigate heat stress. To reduce fertilizer inputs and improve ecological adaptability, we emphasize the enhancement of nutrient use efficiency and photothermal insensitivity. Accordingly, we propose a four‐step modern breeding pipeline covering variation generation, selection, and fixation, with genomic selection and modern advanced technologies integrated for hybrid prediction, and discuss how to solve the trait trade‐off through a three‐phase roadmap.

## 2 Current Maize Breeding Objectives for High Yield

### 2.1 Increasing Plant Density

#### 2.1.1 Optimizing Plant Architecture

**FIGURE 1:**
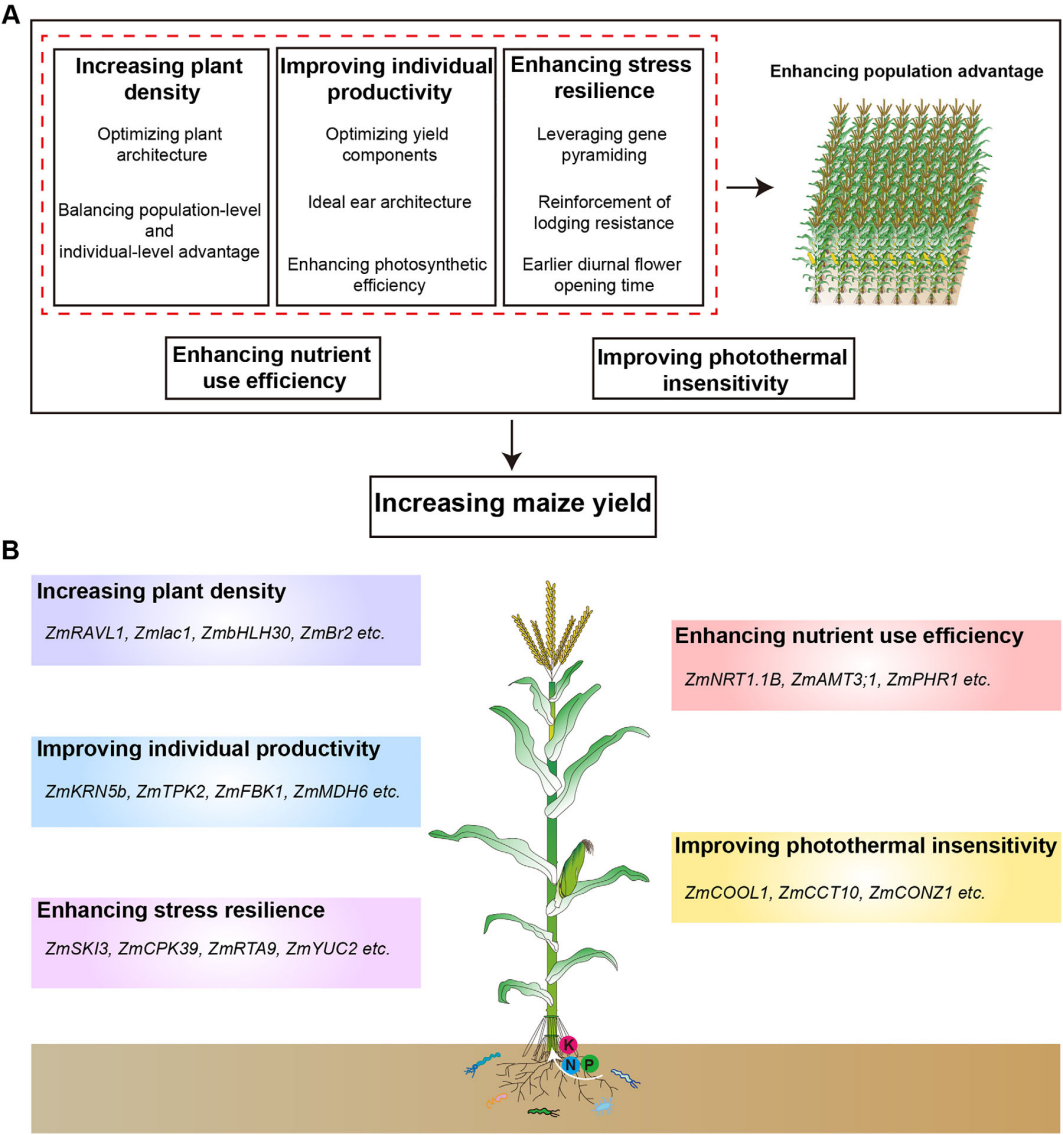
Maize breeding objectives for high yield. (A) Enhancing population advantage to improve maize yield. The core breeding objectives include: increasing plant density by optimizing plant architecture and balancing population‐level and individual‐level advantage; improving individual productivity by optimizing yield components, constructing ideal ear architecture, and enhancing photosynthetic efficiency; and enhancing stress resilience through gene pyramiding, reinforcement of lodging resistance, and advancing diurnal flower opening time. These strategies collectively enhance population advantage under dense planting conditions. Furthermore, improving nutrient use efficiency reduces fertilizer input, while enhancing photothermal insensitivity expands ecological adaptability. Together, these approaches contribute to the continuous increase of maize yield. (B) Genetic pathways supporting the breeding objectives. This figure presents key genes regulating different breeding objectives. For example, *ZmRAVL1* and *Zmlac1* regulate plant architecture and density; *ZmKRN5b* and *ZmMDH6* influence individual productivity; *ZmSKI3* and *ZmCPK39* are involved in stress resilience; *ZmNRT1.1B* and *ZmAMT3;1* modulate nutrient use efficiency; and *ZmCOOL1* and *ZmCCT10* regulate photothermal insensitivity.

Plant architecture is a pivotal determinant of the feasibility and productivity of high‐density planting in maize. An optimized architectural framework enhances tolerance to dense populations while maintaining high photosynthetic efficiency and effectively coordinating source‐sink‐flow relationships within the plant canopy [15, 16, 17]. Among architectural traits, plant height shows a strong inverse relationship with planting density, making reduced stature a desirable characteristic for sustaining yield under high‐density conditions [18, 19, 20, 21]. In addition to plant height, leaf angle plays a critical role in canopy light interception. Moderately erect leaves at the upper canopy help maintain a compact plant architecture while preserving efficient light capture and minimizing mutual shading [22, 23, 24]. Collectively, the defining features of an ideal maize plant architecture for dense planting, therefore, include reduced plant height and ear height, maintenance of moderate leaf angles at different canopy levels, and optimized spatial arrangement of functional leaves. This maize ideal architecture optimizes the vertical distribution of photosynthetically active leaves in the upper canopy, thereby maximizing light interception and assimilate production for grain filling. A growing number of genes have been identified that contribute to density tolerance through modulation of plant architecture. For example, *ZmRAVL1* reduces the leaf angle by regulating endogenous brassinosteroid levels in the collar, leading to a more compact plant architecture [25]. The smart‐canopy architecture gene *Zmlac1* dynamically adjusts leaf angle in response to light signals, facilitating adaptation to high‐density environments [26]. In addition, *ZmbHLH30* and *ZmbHLH155* have been identified as positive regulators of leaf angle development [27]. For plant height control, the *Br2‐Cas9* cassette demonstrates broad applicability and effectiveness in generating an allelic series of *Br2*, enabling fine‐tuned modulation of plant stature across diverse genetic backgrounds [28]. Together, favorable alleles of these architecture‐related genes provide valuable genetic resources for breeding maize varieties with enhanced tolerance to high‐density planting (Figure 1).

#### 2.1.2 Balancing Population‐Level and Individual‐Level Advantage

Studies in Argentina have shown that hybrids with superior biomass and high reproductive partitioning have a lower optimal planting density. Their harvest index declines more significantly with increasing planting density [29]. Similarly, in China, elite maize hybrids with high individual advantage, such as Xianyu335, typically exhibit tall plant stature and high individual plant yield, but require relatively low planting densities to avoid excessive competition. In contrast, MY73 [30, 31] and Kangnongyu8009 [32], which have a weaker individual advantage than Xianyu335 or Zhengdan958, are shorter in stature, better adapted to high‐density planting, and rely on population‐level yield advantages. Gradient planting density experiments identified optimal density ranges of 60 000–67 500 plants ha^−1^ for Xianyu335, 67 500–82 500 plants ha^−1^ for Zhengdan958, and 82 500–97 500 plants ha^−1^ for MY73. As planting density increased, the grain yield of MY73 progressively surpassed that of the other two cultivars and then stabilized with little variation. In contrast, yields of Zhengdan958 and Xianyu335 decreased sharply [33]. These observations suggest that excessive individual advantage constrains planting density. Accordingly, we propose a breeding objective that balances population‐level and individual‐level advantage, thereby mitigating the density‐limiting effect of excessive individual performance and enabling higher planting densities. In maize breeding, blind and excessive pursuit of hybrids with strong individual advantage should be avoided. Thus, maize yield improvement is not inherently dependent on excessive individual advantage. A rational balance between population‐level and individual‐level advantages should be established to achieve optimal coordination under high planting density. By moderately attenuating individual advantage, yield formation can shift from reliance on high individual plant performance toward enhanced population advantage under dense planting, thereby improving density tolerance while maintaining high overall yield (Figure 1).

### 2.2 Improving Individual Productivity

#### 2.2.1 Optimizing Yield Components

Under high‐density planting conditions, further gains in population yield increasingly depend on maintaining or enhancing individual plant productivity [10]. Grain yield in maize is primarily determined by three key components: ear number, kernel number, and hundred‐kernel weight [34, 35]. Increasingly, genes with additive effects can be exploited to increase ear length and kernel row number, two major determinants of kernel number per ear. The cloning and functional characterization of key additive genes, including *ZmKRN5b* [36, 37], *ZmYIGE1* [38], *ZmACO2* [39], and *ZmTPK2* [40], have significantly advanced our understanding of the genetic control of ear development and provide valuable targets for improving individual productivity under dense planting. Superior allelic variations of these genes enable balanced population pressure and individual growth potential, contributing to high and stable maize yield under dense planting. Collectively, per‐plant yield can be effectively maintained or enhanced under high‐density conditions by integrating additive‐effect yield genes (Figure 1).

#### 2.2.2 Ideal Ear Architecture

Attention now turns to the optimization of ear morphology as a means to enhance per‐plant yield. We introduce the concept of an “Ideal Ear Architecture” as a breeding objective aimed at improving per‐plant productivity through optimized ear morphology. Maize ears generally exhibit two principal architectural types: conical and cylindrical. Conical ears taper toward the apex, often resulting in reduced kernel set in the apical region, whereas cylindrical ears maintain a relatively uniform circumference along the cob, supporting more consistent kernel development and a higher total kernel number. Improved apical kernel set in cylindrical ears, together with their superior performance under dense planting, confers both individual‐plant and population‐level yield advantages. Elucidating the genetic basis of ear architecture, therefore, facilitates its targeted optimization in breeding programs. In brief, optimized ear architecture sustains and elevates per‐plant yield under high‐density planting (Figure 1).

#### 2.2.3 Enhancing Photosynthetic Efficiency

Within the maize canopy, upper leaves primarily absorb red and blue wavelengths for photosynthesis, whereas far‐red light is largely transmitted or reflected [41]. High‐density planting markedly reduces the red‐to‐far‐red (R: FR) light ratio in the middle and lower canopy layers, thereby altering the light environment and triggering the shade avoidance response (SAR) [42]. SAR is characterized by increased plant height, altered leaf angle, accelerated flowering, premature leaf senescence, reduced disease resistance, and weakened stem strength that increases susceptibility to lodging, ultimately leading to yield penalties [15, 43, 44]. Some approaches can be employed to enhance photosynthetic efficiency under dense planting. Increasing the effective R: FR ratio in functional leaves is critical for maintaining photosynthetic performance under high‐density planting by reducing sensitivity to SAR. Consistent with this approach, loss‐of‐function mutations in *ZmPIF3s*, *ZmPIF4s*, and *ZmPIF5s* reduce sensitivity to shading [45, 46], while mutation of *ZmDBB2* diminishes responsiveness to low R: FR signals, thus conferring insensitivity to SAR [47]. Additionally, the photosynthetic limitation caused by low light intensity under dense planting can be mitigated by increasing leaf chlorophyll content, enhancing photosynthetic rate, and employing photosynthesis bioengineering. The genes *ZmFBK1* [48], *ZmCAO1* [49], and *ZmPAO* [50] offer targets for improving photosynthetic efficiency through the modulation of chlorophyll content. Knockout of *ZmDapF1* results in an enhancement of ZmMDH6 activity and a concomitant increase in photosynthetic rate [51]. Rubisco can be engineered to enhance CO_2_ affinity without compromising catalytic speed, thereby directly increasing photosynthetic efficiency [52, 53]. Together, these advances contribute to enhanced canopy photosynthetic efficiency, increased assimilate production, and greater dry matter accumulation, thereby supporting stable yield formation under high‐density planting (Figure 1).

### 2.3 Enhancing Stress Resilience

#### 2.3.1 Leveraging Gene Pyramiding

Ear rot, stalk rot, Southern rust, and leaf blight represent major biotic constraints on maize yield worldwide. Besides widespread biotic diseases, maize productivity is also constantly challenged by diverse abiotic stresses such as drought, salinity, and heat stress [54, 55, 56, 57]. Resistance to some biotic and abiotic stresses, particularly ear and stalk rots, is typically governed by multiple genes with small individual effects rather than by single major resistance loci [58, 59, 60]. Single‐gene resistance is often race‐specific and prone to breakdown by the emergence of new pathogen races, as documented for southern corn rust in maize [61, 62]. To achieve durable and broad‐spectrum stress resistance, we propose that commercial hybrids should harbor multiple distinct resistance genes within their genomes, thereby increasing the genetic breadth of overall stress resilience. For instance, the hybrid MY73 possesses five heterozygous disease‐resistance loci, three of which confer dominant resistance, explaining its superior resistance to multiple diseases, including southern corn leaf blight, southern corn rust, and corn head smut. In addition, MY73 also harbors favorable alleles associated with drought and salt tolerance [31]. The continued identification of stress‐resilience‐associated genes, including *ZmSKI3* [63], *ZmCPK39* [64]*, ZmRTA9* [65], *ZmEREB58* [66], *ZmHSF12* [67], and *ZmATG8c* [68], further expands the repertoire of genetic resources available for polygenic resistance breeding toward both biotic and abiotic stress adaptation. Together, these findings highlight the value of pyramiding multiple resistance loci to achieve stable, long‐term stress resistance in high‐density maize production systems (Figure 1).

#### 2.3.2 Reinforcement of Lodging Resistance

Under high‐density planting conditions, intensified competition among neighboring plants often compromises root and stalk development, increasing susceptibility to lodging. Lodging has a pronounced negative impact on yield, with a reported 1% increase in lodging rate resulting in a yield loss of approximately 108 kg/hm^2^ [69]. Maize lodging is categorized into stalk lodging and root lodging [70]. Stalk lodging resistance depends on structural integrity and biochemical composition, particularly the polymers cellulose and lignin. Cellulose provides mechanical strength, whereas lignin enhances cell wall hardness and stability, thereby improving lodging resistance [71, 72, 73, 74, 75]. Therefore, stalk lodging resistance can be enhanced by optimizing cellulose and lignin content to improve stalk flexibility. Brace root angle is a key determinant of anchorage strength and lodging resistance in maize [76, 77]. While steeper brace root angles generally provide better mechanical support, the optimal angle likely depends on soil conditions and plant density [78]. Future research should aim to define the ideal brace root architecture through biomechanical modeling. Engineering brace roots architecture is expected to maximize mechanical stability and anchorage strength under dense planting conditions. At the molecular level, *ZmYUC2* and *ZmYUC4* are specifically expressed at the tips of maize brace roots and contribute to localized auxin biosynthesis, thereby influencing root gravitropism and regulating growth angle [78]. Exploiting allelic variation in these and related genes to modulate root gravitropic responses offers a promising strategy to optimize brace root penetration angles and enhance lodging resistance in high‐density maize systems. Overall, stalk lodging and root lodging can be effectively mitigated by improving stalk flexibility and optimizing brace root architecture, respectively (Figure 1).

#### 2.3.3 Earlier Diurnal Flower Opening Time

The frequency and severity of maize yield losses caused by high‐temperature stress have increased markedly over the past five decades, with an estimated average yield reduction of approximately 7.4% per 1°C increase in temperature [79]. Although heat stress affects maize throughout its life cycle, the flowering stage is particularly vulnerable [80, 81, 82, 83]. During this period, elevated temperatures negatively impact pollen production, pollen viability, and silk receptivity, ultimately leading to poor pollination and reduced kernel set [84, 85]. When temperatures exceed 36°C, anther dehiscence is often incomplete, resulting in reduced pollen release and viability, and pollen viability is almost entirely lost at temperatures above 38°C [86]. In most maize hybrids, pollen shedding begins in the late morning and peaks toward midday, coinciding with the daily rise toward maximum field temperatures. Therefore, it is possible to avoid heat stress damage by ensuring that pollen shedding begins sufficiently early and peaks before the onset of daily maximum temperatures in maize. This temporal shift enables pollen shedding and fertilization to occur under cooler morning conditions, effectively avoiding daily peak temperatures and thereby preserving pollen viability and fertility [87]. As a result, earlier diurnal flowering confers a more stable kernel set under heat stress. Although the genetic regulation of diurnal flower‐opening time in maize remains poorly understood, existing evidence suggests that this trait represents a promising and underexploited target for mitigating heat‐induced reproductive failure. Insights from rice provide valuable clues: cell wall pectin methylesterification has been shown to regulate daily flower‐opening time [88], natural variation in *OsMYB8* underlies differences in flower opening time between subspecies [89], and the OsMYC2‐JA feedback loop controls diurnal flower opening through cell wall remodeling [90]. These findings highlight conserved regulatory mechanisms that may inform the identification and manipulation of analogous pathways in maize (Figure 1).

### 2.4 Enhancing Nutrient Use Efficiency

Low nitrogen and phosphorus use efficiencies [91, 92, 93], together with suboptimal potassium utilization [94, 95], remain major constraints on agricultural productivity and contribute substantially to environmental pollution. Elite cultivars are often highly dependent on fertilizer inputs yet exhibit relatively low nutrient use efficiency [96, 97]. As planting density increases, nutrient demand per unit area rises sharply, frequently necessitating additional fertilizer application to sustain high yields. Consequently, increasing crop yields without further escalating fertilizer inputs has emerged as a central challenge in modern agriculture [98, 99]. To address this issue, it is necessary to enhance the root nutrient acquisition capacity and internal nutrient translocation efficiency. Multiple genes involved in nutrient uptake, transport, and signaling, including *ZmNRT1.1B* [100], *ZmAMT3;1* [101], *ZmNLP8* [102], *ZmPHR1* [103], *ZmID1* [104], *ZmNPF7.10* [105], have been identified as key regulators of nutrient absorption and movement, providing a strong genetic foundation for improving nutrient use efficiency in maize. Besides, root activity and nutrient availability can be further enhanced by strengthening interactions between maize roots and beneficial soil microorganisms [106]. Through the secretion of diverse root exudates, plants actively recruit beneficial microbes that improve nutrient acquisition and translocation, while simultaneously enhancing resistance to both abiotic and biotic stresses [101, 107, 108]. Looking ahead, targeted genetic optimization of both plant and microbial genomes offers a promising avenue for amplifying these beneficial interactions and enabling multifunctional improvements in agroecosystems [109]. Together, integrating improved nutrient acquisition, optimized plant–microbe interactions, and high‐density–adapted breeding objectives will make it possible to achieve high yield and enhanced stress resistance without increasing fertilizer inputs. This approach supports the development of green agriculture, protects ecological systems, and promotes long‐term agricultural sustainability (Figure 1).

### 2.5 Improving Photothermal Insensitivity

Maize is a short‐day crop that originated in tropical and subtropical regions and consequently exhibits high sensitivity to low temperatures [110, 111, 112, 113]. Through extensive artificial selection, temperate maize germplasm has largely reduced photoperiod sensitivity [114], enabling successful cultivation at higher latitudes. However, the strong photoperiod sensitivity retained by many tropical and subtropical maize accessions continues to limit their direct utilization in temperate regions. Moreover, both photoperiod and temperature act as major environmental constraints that restrict the ecological adaptation range of otherwise elite varieties, thereby hindering their large‐scale deployment [115, 116]. Expanding the adaptive range of maize cultivars has therefore become a key objective in modern breeding programs. Recent progress has identified several maize genes associated with low‐temperature tolerance, such as *ZmCOOL1* [116], *ZmBARK1* [117], *ZmRR1* [118], and *ZmTIP4;3* [119], as well as key regulators of photoperiod response such as *ZmFKF1a* [120], *ZmCCT10* [115], and *ZmCONZ1* [121]. The identification and deployment of favorable alleles at these loci have facilitated maize cultivation in high‐latitude and high‐altitude regions and provide powerful genetic tools for expanding the adaptive range of elite hybrids (Figure 1).

## 3 The Four‐Step Modern Breeding Pipeline

### 3.1 Generate Variation

#### 3.1.1 Identification of Elite Natural Variations from Wild Relatives and Landraces

**FIGURE 2:**
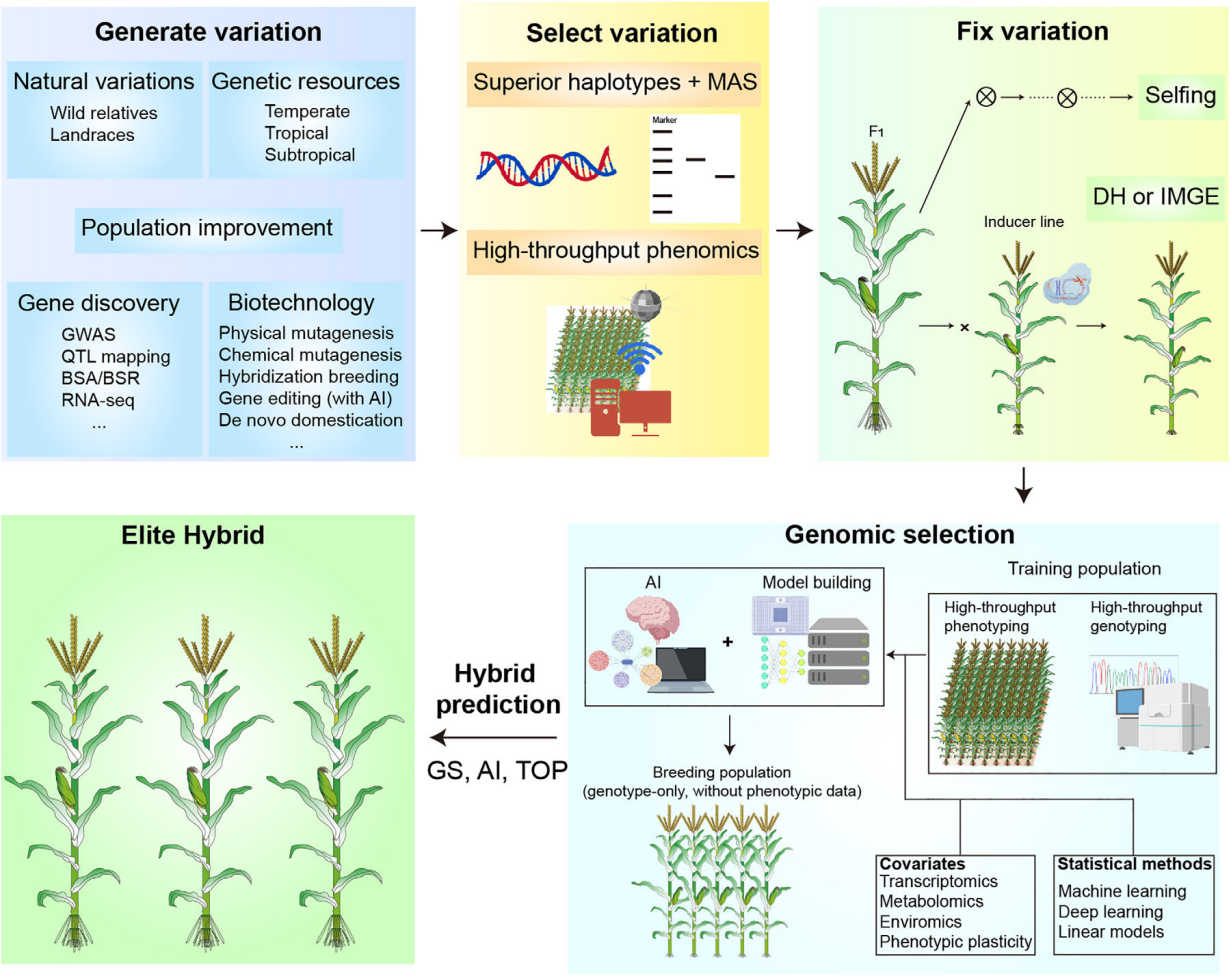
The four‐step modern breeding pipelines. This figure illustrates four key components: variation generation, variation selection, variation fixation, and genomic selection for hybrid prediction. Variation generation. Genetic variation can be created by mining superior alleles from wild relatives and landraces, integrating genetic resources from temperate, tropical, and subtropical regions, breaking genetic linkage drag through population improvement, identifying key genes (e.g., via GWAS, QTL mapping, BSA/BSR, RNA‐seq), and applying diverse biotechnological approaches (e.g., mutagenesis, hybridization breeding, gene editing, de novo domestication). Variation selection. Selection is achieved through superior haplotypes combined with marker‐assisted selection, together with high‐throughput phenomics technologies. Variation fixation. Homozygous fixation of genetic variation and the generation of genotypically stable materials are accomplished by successive selfing, doubled haploid technology, and the IMGE system. Genomic selection for hybrid prediction. A prediction model is built using a training population that includes both genotypic and phenotypic data, incorporating covariates (e.g., transcriptomics, metabolomics, enviromics, phenotypic plasticity) and statistical models (e.g., linear models, machine learning, deep learning). This approach enables accurate prediction of hybrid phenotypes using only genotypic data from the breeding population, without requiring their phenotypic data. Coupled with techniques such as TOP (selection of optimal parents) and AI, genomic selection allows efficient prediction of elite hybrids, thereby accelerating the breeding process. Some elements were created with BioGDP.com [206].

Maize was domesticated from teosinte, a wild progenitor with exceptional stress resilience and considerable genetic research potential [122, 123, 124, 125, 126, 127, 128]. Several wild relatives of maize retain valuable adaptive traits. For example, *Zea nicaraguensis* and *Zea luxurians* could be utilized to improve waterlogging resistance [129, 130]. Quantitative trait locus (QTL) mapping has further revealed the breeding value of wild germplasm. Two major QTLs controlling leaf angle, designated *UPA1* and *UPA2*, were identified in introgression line populations derived from crosses between W22 and the CIMMYT 8759 accession [25]. In addition, *TSH4*, a key regulator of maize plant architecture, was identified using the teosinte nested association mapping population [131]. These findings underscore the presence of numerous favorable alleles in wild relatives that have been lost or underutilized during modern breeding. Such genetic resources are critical not only for broadening the narrow genetic base of elite maize but also for providing a foundation to generate abundant naturally favorable variants (Figure 2).

Owing to their long‐term selection under local conditions, maize landraces constitute a key genetic reservoir for generating variation [132, 133, 134]. During the early twentieth‐century transition from landraces to improved hybrids, many favorable alleles were likely lost as a consequence of genetic drift or linkage with undesirable traits [135, 136, 137, 138]. As a result, landraces remain an important source of genetic diversity for modern breeding, particularly for traits associated with tolerance to adverse environmental conditions [139]. The successful exploitation of landrace germplasm by Pioneer Hi‐Bred illustrates this potential: Argentinian landraces were used to establish the novel female pool “Maize Amargo,” from which elite inbred lines such as B96 and PHG39 were derived [140], substantially improving breeding efficiency and varietal competitiveness. Similarly, in China, the PA, PB, and X groups were developed through sustained utilization of landrace germplasm [141, 142, 143, 144, 145, 146, 147]. Among these, Tangsipingtou germplasm is characterized by compact plant architecture and upright leaves, and hybrids derived from this background often display strong tolerance to medium and high planting densities [148]. Collectively, these examples demonstrate that landraces contain elite alleles conducive to generating genetic variation (Figure 2).

#### 3.1.2 Integrating Diverse Ecological Genetic Resources

A complementary method for generating variation is the construction of new breeding populations that integrate the distinct advantages of temperate, tropical, and subtropical genetic resources [149, 150, 151]. The substantial genetic divergence among these ecological groups means that gene flow across regions can broaden genetic backgrounds, compensate for genetic deficiencies, and enhance combining ability. However, the strong photothermal sensitivity of exotic germplasm presents major challenges, including asynchronous flowering between male and female inflorescences, yield instability, and prolonged growth duration, which limit its direct deployment across diverse environments [152]. To overcome these constraints, we can strategically integrate favorable alleles from germplasm of diverse ecological regions into local breeding pools to create new genetic variation. Hybrid breeding is commonly used to develop new germplasm, with progeny selection typically conducted via the pedigree method. Two principles are critical for effective selection. First, segregating populations must be sufficiently large to maximize the probability of recovering superior individuals and to expand the scope of artificial selection. Second, selection intensity should be deliberately increased under relevant stress conditions (Figure 2).

#### 3.1.3 Breaking Linkage Drag by Population Improvement

In breeding, the tight physical linkage (genetic linkage) between a favorable allele and an unfavorable one often results in linkage drag, where undesirable traits are co‐inherited with beneficial ones [153]. Maize population improvement represents a powerful and complementary method for breaking linkage drag to generate new variation. This approach relies on artificial selection and targeted intervention to disrupt the Hardy‐Weinberg equilibrium within breeding populations. Through recurrent selection, linkage between favorable and unfavorable alleles is progressively broken, thereby enhancing recombination among beneficial loci and increasing the frequency of advantageous alleles at the population level [154, 155]. As a result, adaptive traits from exotic germplasm can be gradually introgressed into local germplasm pools, thereby broadening genetic diversity and enhancing overall breeding potential. Numerous successful examples worldwide demonstrate the effectiveness of population improvement. For example, the Iowa Stiff Stalk Synthetic population, initiated in 1939, has undergone continuous improvement and served as the genetic foundation for elite inbred lines such as B73, B14, and B121 [156, 157]. Similarly, the Suwan 1 population, developed and steadily improved since the 1960s, has become a key germplasm resource in Asia and the Americas [158, 159]. In China, the general combining ability was effectively improved in the Yuzong 5 population by half‐sib recurrent selection and half‐sib reciprocal recurrent selection. This improvement successfully developed multiple elite inbred lines from this population, such as Yu82, Xinzi534, Chuan341, and J‐5 [160, 161]. Building upon these proven cases of population improvement, we propose that constructing new breeding populations from existing elite inbred lines provides an effective means to generate novel variation that meets specific breeding objectives while simultaneously expanding the genetic base (Figure 2).

#### 3.1.4 Gene Discovery for Targeted Trait Improvement

A range of genetic dissection approaches, such as genome‐wide association study (GWAS), quantitative trait locus analysis, bulked segregant analysis (BSA), and bulked segregant RNA sequencing (BSR), enable the exploration of functional genes and the optimization of breeding populations to support targeted genetic improvement. Although several functionally characterized genes have been reported to support the achievement of breeding objectives, numerous genes regulating key agronomic traits remain poorly understood, requiring further exploration and identification of novel functional genes to facilitate precise breeding. Genes such as *Zmlac1* can be utilized to optimize plant architecture, whereas increased planting density requires moderate attenuation of individual advantage. The introduction of additive genes regulating yield components and photosynthetic efficiency in both parental lines can enhance individual productivity, while the genetic basis underlying ideal ear architecture remains largely uncharacterized. Genes such as *ZmSKI3* have been applied to improve stress tolerance, but only a limited number of genes controlling brace root architecture and diurnal flower opening time have been identified. Several genes associated with nutrient utilization and photothermal responsiveness have been identified, but further discovery of pivotal genes is still needed to improve nutrient uptake and broaden ecological adaptability across diverse environments (Figure 1B). In summary, ongoing efforts to uncover novel functional genes are fundamental to advancing targeted trait improvement and realizing the proposed breeding objectives (Figure 2).

#### 3.1.5 Biotechnology

Physical and chemical mutagenesis can directly induce random genetic variation and is generally unrestricted by relevant policies. Radiation and chemical reagents are utilized to trigger mutations, and the combination of low‐cost sequencing enables the identification of variations in specific genes [162]. Technical methods, including hybridization, backcrossing, and distant hybridization, can pyramid parental traits to generate new variation. Genome editing represents a revolutionary technology for crop genetic improvement that can precisely modify genomic sequences and induce targeted mutations [163]. In recent years, increasing attention has been directed toward de novo domestication as a complementary strategy to traditional breeding [164]. De novo domestication can circumvent the lengthy cycles of hybridization and selection inherent to conventional domestication, enabling the rapid and precise conversion of wild species into agronomically useful crops [165, 166, 167]. With continued advances in biotechnology–particularly genome editing and functional genomics–it is now feasible to rapidly domesticate teosinte while retaining or reintroducing superior adaptive alleles that were lost during early domestication. Together, these biotechnological methods provide powerful and versatile tools for accelerating genetic variation creation and precise trait modification (Figure 2).

### 3.2 Select Variation

During the selection of favorable variants, approaches such as superior haplotype analysis coupled with marker‐assisted selection (MAS) can accelerate the identification of beneficial alleles, thereby enabling targeted and precise selection and pyramiding of target traits while broadening the genetic background. Meanwhile, high‐throughput phenotyping systems can continuously capture multidimensional, dynamic, and large‐scale phenotypic information across entire populations throughout the growth cycle. This technology enables the precise quantification of complex agronomic traits under variable field and environmental conditions, and objectively evaluates the phenotypic performance of individual plants. It effectively improves the screening efficiency of favorable genetic variations and provides reliable phenotypic support for the accurate identification and rational utilization of elite germplasm resources [168, 169]. However, plant phenomics is constrained by three core bottlenecks: the absence of standardized frameworks, insufficient adaptability of analytical models, and limited integration between molecular phenotypes and biological models [170]. Accordingly, breaking these limitations will further unlock the potential of high‐throughput phenotyping and better serve the efficient screening and utilization of favorable genetic variations in maize breeding.

### 3.3 Fix Variation

Following the selection of superior genetic variants, the breeding process advances to the fixation of these favorable alleles into a genetically stable state. This critical step ensures that beneficial genotypes are preserved and maintained in a homozygous state for stable heritability across generations. Traditional fixation is achieved through repeated cycles of self‐pollination, which gradually eliminates heterozygosity and stabilizes the selected genotype over multiple generations. However, this conventional method is labor‐intensive, time‐consuming, and requires multiple generations to achieve homozygosity. In recent years, double haploid (DH) breeding–based on in vivo haploid induction–has become a cornerstone of modern maize improvement, enabling the rapid production of completely homozygous lines and substantially increasing breeding efficiency [171, 172, 173]. The Stock6 line was the first haploid inducer identified in maize, and its derivatives are now widely deployed in breeding programs worldwide [173, 174]. Haploid‐Inducer Mediated Genome Editing (IMGE) has been successfully applied to engineer smart canopy architecture in multiple inbred lines, including HI3, B104, PH207, and OSL476 [26, 175], enabling the rapid creation of density‐tolerant germplasm. Collectively, these approaches facilitate the efficient fixation of favorable variation and ensure the genetic stability of elite breeding materials (Figure 2).

### 3.4 Genomic Selection and Hybrid Prediction

The genetic mechanism underlying heterosis remains elusive [141]. In conventional maize breeding, this forces breeders to generate numerous hybrid combinations and conduct extensive field evaluations to identify superior hybrids, making hybrid evaluation a critical bottleneck. Recent advances in technology and interdisciplinary integration offer the potential to overcome this limitation. Genomic selection (GS) was proposed, which uses phenotypic and genotypic data to train machines, applies corresponding statistical models to evaluate the genetic effects, and obtains the breeding values of individuals to select superior maize varieties [176]. High‐throughput population genetics, plant phenomics, low‐cost genotyping, together with data technology and artificial intelligence (AI), collectively empower genomic selection to better harness crop genetic potential [177, 178, 179, 180, 181, 182]. For instance, a joint analysis of GWAS, GS, and genomic evaluation of parental inbred lines was performed to accelerate the breeding of superior maize hybrids [183, 184]. The GEFormer model, which integrates a gating multilayer perceptron and linear attention mechanisms, delivers enhanced prediction performance in actual maize breeding scenarios [185]. These modern technologies are clearly propelling progress in contemporary breeding. In the future, the influx of large‐scale biological data poses a major challenge for researchers and existing analytical frameworks. This necessitates the development of advanced data technology for effective analysis and mining [186, 187]. Artificial intelligence is renowned for data analysis and pattern recognition to ensure selection accuracy and boost breeding efficiency [188, 189]. Taken together, these advances provide a powerful breeding approach, in which a training population comprising both genotypic and phenotypic data is used to construct statistical or machine learning models that establish robust associations between genotypes and phenotypes. Such models can subsequently be applied to breeding populations with only genomic profiling data, enabling accurate selection of elite hybrids without prior field evaluation. GS integrated with diverse complementary technologies, such as the target‐oriented prioritization (TOP) [190] and AI, provides a promising solution to break through this bottleneck and improve breeding efficiency (Figure 2).

## 4 Concluding Remarks and Perspectives

Against the backdrop of rapid population growth, climate change, farmland degradation, and the increasing severity of pests and diseases [191, 192, 193], there is an urgent demand for elite maize hybrids. To meet this demand, we have proposed a set of breeding objectives to improve yield potential while enhancing resilience under future global challenges. The trait package proposed above is not intended as a blueprint for a single “super variety” that simultaneously incorporates all features. Rather, it serves as a research agenda to systematically evaluate and resolve the inherent trade‐offs, environment‐dependent behaviors, and practical constraints that any real‐world breeding program must confront. We recognize that inherent genetic and phenotypic trade‐offs are inevitable when pursuing simultaneous improvement of such a broad suite of traits, even with synergistic selection. For instance, pyramiding numerous favorable alleles across the genome—essential for integrating multiple synergistic traits—can introduce linkage drag and reduce breeding efficiency, as tightly linked deleterious alleles may be co‐introgressed and excessive target loci can extend the breeding cycle [194]. We hope that the distinctive breeding objectives proposed herein, particularly the balance between population‐level and individual‐level advantages, can provide reasonable guidance for density optimization among maize breeders across diverse ecological regions. Breeding practices should refrain from the excessive pursuit of superior individual performance; instead, moderate attenuation of individual advantage can support rational increases in planting density. Once the regional planting density achieves its optimal planting level, further yield gains rely on the improvement of individual productivity, as observed in the U.S. Midwest [10]. This perspective aims to provoke extensive discussion on how to balance the trade‐off between reducing individual‐level advantage to raise planting density and improving individual productivity for enhanced population advantage. Nonetheless, with the progressive advancement of modern breeding technologies, the inherent trade‐offs among key agronomic traits are expected to be effectively alleviated in subsequent genetic improvement programs. To make this agenda actionable, we propose a three‐phase roadmap grounded in the analyses summarized in Tables 1 and 2.

**TABLE 1:** Synergies, conditional compatibility, and inherent trade‐offs among the proposed breeding traits in maize.

Trait / Objective	Synergistic with	Conditionally compatible with	Inherent trade‐off / antagonistic with	Proposed decoupling strategy
Optimizing plant architecture (compact architecture) (plant height and leaf angle)	Enhanced photosynthetic efficiency (less mutual shading); higher planting density	Lodging resistance (synergistic under high density; neutral at low density)	Reduced individual productivity	Use modular breeding to realize modular and targeted optimization
Balancing population‐level and individual‐level advantage	Higher density tolerance; population‐level yield	—	Individual productivity (if over‐reduced)	Moderate reduction, not elimination
Optimized ear architecture (cylindrical) and yield components	Higher kernel number per ear	Earlier flowering (neutral to synergistic in most environments)	—	None needed; generally additive
Enhanced photosynthetic efficiency	Compact architecture (synergistic in canopy); higher biomass	Earlier flowering (if longer grain filling needed)	Earlier flowering in long‐season environments	Temporal decoupling: enhance photosynthesis without accelerating senescence
Leveraging gene pyramiding	Broad‐spectrum durable resistance	—	Pyramiding many loci may cause linkage drag	Use MAS/GS to minimize linkage drag; phased pyramiding
Reinforcement of lodging resistance	Lodging resistance under high density	Compact architecture (compatible)	Mechanical harvest (if lodging resistance is excessively strong)	Optimize stalk flexibility and brace root number/angle; test with harvest equipment
Earlier diurnal flower opening time	Heat stress escape	—	Grain yield in long‐season, high‐input systems (yield penalty from shorter growing period)	Environment‐specific deployment: target heat‐prone, short‐season regions only
Enhanced nutrient use efficiency	Reduced fertilizer input; environmental sustainability	Broader adaptation (synergistic)	—	None; generally additive
Improved photothermal insensitivity (broader adaptation)	Stable performance across latitudes	—	Specific adaptation (loss of local specialization)	Use environment‐stratified breeding; maintain some local adaptation alleles

**TABLE 2:** Feasibility bottlenecks and phased readiness of proposed breeding traits.

Trait	Phenotyping throughput	Regulatory status (gene editing)	Agronomic infrastructure requirement	Readiness phase
Optimizing plant architecture (compact architecture) (plant height and leaf angle)	High (ruler, protractor, drones)	SDN‐1 (low barrier in many countries)	None (standard equipment)	Short‐term (3–5 yr)
Balancing population‐level and individual‐level advantage	High (field measurement)	SDN‐1	None	Short‐term
Optimized ear architecture (cylindrical) and yield components	Medium (manual or image‐based)	Natural variation / MAS (no editing needed)	None	Short‐term
Enhanced photosynthetic efficiency	Low (gas exchange is low‐throughput; need spectral proxies)	SDN‐2 (moderate barrier)	None	Medium‐term (5–10 yr)
Leveraging gene pyramiding	Medium (disease scoring; molecular markers)	No editing needed (natural alleles)	None	Short‐term
Reinforcement of lodging resistance	Low (root excavation is destructive; need CT or field phenotyping)	SDN‐2 (e.g., ZmYUC2/4 edits)	Potential harvest modification if roots too large	Long‐term (>10 yr)
Earlier diurnal flower opening time	Medium (time‐lapse imaging)	Unknown (regulatory status uncertain)	None	Medium‐term
Enhanced nutrient use efficiency	Medium (tissue N content; isotopic methods)	SDN‐1 / natural variation	Precision fertilization may help but not required	Short‐ to medium‐term
Improved photothermal insensitivity (broader adaptation)	Medium (multi‐location trials)	SDN‐1 (e.g., *ZmCCT10* edits)	Multi‐location testing infrastructure	Medium‐term

*Note* on regulatory classification: SDN‐1 (small indels without template) is regulated as conventional breeding in some countries (e.g., US, Japan) but not in others (e.g., EU). SDN‐2/3 face stricter oversight.

Short‐term (3–5 years): Traits that are phenotypable at high throughput, face minimal regulatory hurdles (Site‐Directed Nuclease 1 (SDN‐1), SDN‐1 edits [195] or natural variation), and require no major infrastructure changes. These include optimizing plant architecture (via *ZmRAVL1* or *Zmlac1* selection), balancing population‐level and individual‐level advantage (moderate attenuation of individual performance), optimizing ear architecture and yield components (via gene discovery or additive genes), and gene pyramiding (pyramiding of natural alleles). These traits can be immediately integrated into ongoing breeding programs using superior haplotypes, marker‐assisted selection, and simple genome editing.

Medium‐term (5–10 years): Traits that require resolved genotype‐by‐environment interaction (G×E) relationships, moderate regulatory approval, or improved phenotyping. These include enhanced photosynthetic efficiency (needing better field phenotyping), earlier diurnal flower opening time (environment‐dependent benefit), enhanced nutrient use efficiency (multi‐location validation), and improved photothermal insensitivity (requires testing across latitudes). For these traits, we recommend environment‐stratified deployment rather than universal application.

Long‐term (>10 years): Traits that need breakthroughs in phenotyping (e.g., stalk flexibility and brace roots, which currently require destructive sampling), relaxed regulation (SDN‐2 [196] or transgene‐like edits), or agronomic system redesign (e.g., modified harvesters for brace‐root‐enhanced stalks). These are high‐reward but high‐effort targets that should be pursued through long‐term investment in enabling technologies.

Critically, we treat G×E not as a statistical nuisance but as a design feature. For each context‑dependent trait, we explicitly define the Target Population of Environments (TPEs) in which it is beneficial versus those where it is neutral or detrimental—such as the Huang‐Huai‐Hai summer maize region versus the northern spring maize region of China. A related concept is phenotypic plasticity—the ability of a single genotype to produce different phenotypes in response to environmental cues [197, 198, 199, 200]. Phenotypic plasticity itself is under genetic control [201]; different genotypes can exhibit different degrees of plasticity in response to the same environmental change. It is the biological basis of G×E: when different genotypes display different plastic responses, G×E emerges at the population level. In crop improvement, plasticity has often been minimized to achieve high and uniform yield. However, harnessing adaptive plasticity can help address G×E challenges. This distinction should be integrated into TPE‐specific design rules. To resolve inherently antagonistic trait pairs—e.g., reduced individual advantage versus traditional hybrid vigor, or earlier flowering versus long‑season yield potential—we propose three decoupling strategies: (i) modular breeding (deploying different trait bundles for different TPEs); (ii) temporal separation (e.g., enhancing photosynthesis without accelerating senescence); and (iii) tissue‑specific expression where applicable.

We recognize that implementing this TPE‑based modular strategy may increase operational complexity and cost, particularly for phenotyping, multi‑environment trials, and specialized trait bundles. However, two considerations help mitigate this concern. First, the declining costs of high‑throughput phenotyping and genotyping are making TPE‑specific selection increasingly accessible. Second, we propose reserving modularization for a small set of high‑impact, environment‑sensitive traits (e.g., flowering time, disease resistance, and drought tolerance), while most agronomic traits remain under a single, broadly adapted module. Thus, modular breeding is best viewed as a targeted complement to existing pipelines, not a wholesale replacement.

Looking beyond the current framework, we envision a practical roadmap to move G×E from description to prediction to design. In the short term, routine deployment of multi‑environment genomic selection with envirotyping covariates can help predict G×E patterns and reduce reliance on extensive field testing. In the medium term, biologically informed G×E models—incorporating known major genes such as *ZmCCT10* for photoperiod sensitivity and *ZmMDH6* for photosynthetic efficiency—can improve prediction accuracy and model interpretability without requiring complete mechanistic knowledge. In the long term, active envirotyping, using controlled environments to deliberately induce and characterize G×E, could accelerate mechanistic understanding and enable environment‑tailored design. The ultimate goal is not to eliminate G×E—which is biologically inherent—but to transform it from a source of uncertainty into a predictable, designable, and actionable feature of modern breeding systems.

Finally, we re‐emphasize three feasibility bottlenecks that the community must address collectively. First, phenotyping throughput remains a core limitation [170]—particularly for root traits (e.g., brace roots), photosynthetic efficiency, and diurnal flowering dynamics. Investment in automated field phenotyping (e.g., drones, light detection and ranging system, thermal imaging) and standardized protocols is urgently needed [168, 202]. Second, regulatory heterogeneity for genome‐edited crops across major agricultural economies (e.g., the US, Brazil, China, EU, Japan) creates uncertainty for international germplasm exchange [203, 204, 205]. We encourage harmonization efforts and note that several proposed traits (e.g., *Br2* edits, *lac1* edits) fall into the SDN‐1 category, which is the least restricted in most jurisdictions. Third, agronomic infrastructure—including narrow‐row planters for compact hybrids and modified combine headers for brace‐root‐enhanced lodging resistance—may require coordinated development with agricultural engineering. These are not insurmountable barriers but rather explicit challenges that should guide research prioritization.

In summary, this perspective does not claim that all proposed traits can or should be simultaneously deployed in all environments. Instead, we offer a structured framework (Figures 1 and 2)—including trait stratification (Table 1), feasibility mapping (Table 2), and a phased roadmap—to help researchers and breeders navigate the trade‐offs, G×E interactions, and bottlenecks that are inevitable in the pursuit of high‐density and stress‐resilient maize.

## Author Contributions

J.T., H.Z., and Y.C. conceived the manuscript. X.L. drafted the manuscript. X.J. and X.Z prepared the figures. J.T., D.D., H.Z., Y.C., X.Z., Z.Z., Y.W., and W.L. revised and proofed the manuscript. X.Z. provided important support for the design of modern maize breeding systems, especially in the conceptual framework of genomic selection. All authors read and approved the contents of this paper.

## Conflicts of Interest

The authors declare no conflicts of interest.

## Data Availability

The data that support the findings of this study are available from the corresponding author upon reasonable request.
